# Lessons from behavioral lateralization in olfaction

**DOI:** 10.1007/s00429-021-02390-w

**Published:** 2021-10-01

**Authors:** Matthias Cavelius, Théo Brunel, Anne Didier

**Affiliations:** 1 Lyon Neuroscience Research Center (CRNL), Neuropop Team, Lyon, France; 2 CNRS 5292, Inserm 1028, Lyon 1 University, Lyon, France

**Keywords:** Olfaction, Behavior, Brain lateralization

## Abstract

Sensory information, sampled by sensory organs positioned on each side of the body may play a crucial role in organizing brain lateralization. This question is of particular interest with regard to the growing evidence of alteration in lateralization in several psychiatric conditions. In this context, the olfactory system, an ancient, mostly ipsilateral and well-conserved system across phylogeny may prove an interesting model system to understand the behavioral significance of brain lateralization. Here, we focused on behavioral data in vertebrates and non-vertebrates, suggesting that the two hemispheres of the brain differentially processed olfactory cues to achieve diverse sensory operations, such as detection, discrimination, identification of behavioral valuable cues or learning. These include reports across different species on best performances with one nostril or the other or odorant active sampling by one nostril or the other, depending on odorants or contexts. In some species, hints from peripheral anatomical or functional asymmetry were proposed to explain these asymmetries in behavior. Instigations of brain activation or more rarely of brain connectivity evoked by odorants revealed a complex picture with regards to asymmetric patterns which is discussed with respect to behavioral data. Along the steps of the discussed literature, we propose avenues for future research.

## Introduction

Behavioral and brain functional asymmetry is now a well-documented evidence across phylogeny, from humans (Broca 1865; Dax 1865; Gazzaniga 2005; Manning and Thomas-Antérion 2011) to other vertebrates and non-vertebrate species as recently reviewed (Vallortigara and Rogers 2020; Güntürkün et al. 2020). Lateralized behaviors go far back in phylogeny, since asymmetry in feeding behavior was reported in a Paleozoic reptile (Reisz et al. 2020). In humans, initial discoveries by Broca in brain-lesioned aphasic patients, followed by the studies in commissurectomized, split-brain patients provided evidence that the two sides of the brain sub-serve different functions, such as language or visuo-spatial cognition for the left and right hemispheres respectively (Volz and Gazzaniga 2017). Brain imaging studies provided more evidence of lateralization of cognitive functions and recently, a large-scale analysis showed that brain activity was lateralized in a comprehensive set of cognitive domains. Furthermore, the lateralized activation patterns were used to reveal a low-dimension organization of the main brain functions across distinct axes including perceptive/motor, communication, decision making and emotion (Karolis et al. 2019). Left–right asymmetry in the organization of cortical functional connectivity at rest was also reported and linked to verbal ability for the sub set of brain regions involved in language (Gotts et al. 2013). Thus, lateralized brain activity may represent a fundamental feature of behavior control and it has been further suggested that brain lateralization could confer some advantages in specific behavioral tasks, such as food search, predator survey, or social communication (Fabre-Thorpe et al. 1993; McGrew and Marchant 1999; Güntürkün et al. 2000; Rogers 2000; Dadda and Bisazza 2006) or song production in birds (Nottebohm 1970). However, there is no unified theory yet and the function of brain lateralization remains largely a mystery (Rogers 2014; Güntürkün et al. 2020; Vallortigara and Rogers 2020).

The brain is fed by sensory information and any asymmetry in sensory reception, sampling or processing may play a large part in overall brain lateralization. Hence, a better understanding of asymmetric sensory processing may shed light onto the function of lateralization. Behavioral studies in different species documented the concept of sensory dominance corresponding to a best sensory channel whose stimulation leads to better perceptive performance than that of the other side. In humans, eye dominance has been largely studied at the behavioral level (Chaumillon et al. 2015) and was found to depend on whether one measures visual acuity, the best eye to achieve a monocular task or the eye dominating when two conflicting stimuli are presented to the two eyes (Seyal et al. 1981; Mendola and Conner 2007). In other species, such as fishes (Facchin et al. 1999; Bisazza and de Santi 2003), birds (Mench and Andrew 1986; Zucca and Sovrano 2008) or non-vertebrate species, such as cuttlefish (Schnell et al. 2016) or Crustacea (Daly et al. 2017), an eye performed better or was actively preferred by the animal over the other one to achieve specific tasks. For instance, chicks preferentially use their left eye to deal with a potential predator’s attack (Facchin et al. 1999; Bisazza and de Santi 2003; Zucca and Sovrano 2008), and their right eye to perform a fine visual discrimination task (Rogers 2014) or for conspecific recognition (Zucca and Sovrano 2008; Guo et al. 2009). Taking advantage of the almost complete decussation at the optic chiasm and of the lack of corpus callosum in the visual pathway in chicks (Cowan et al. 1961), resulting in a contralateral processing of the visual inputs, it can be concluded that the left and right hemispheres are specialized in processing visual stimuli bearing distinct ecological significance. In the auditory system, where contralateral projections of sensory information dominate, the calls emitted by the newborn rats out of the nest triggered the maternal response (collect the babies and bring them back to the nest), only when processed by the right ear, *i.e.* the left hemisphere (Ehret 1987). Such lateralized stimulus processing was also reported in acoustic recognition of conspecifics in California sea lions (Böye et al. 2005), European starlings (George et al. 2002) or Japanese macaques (Petersen et al. 1978) (for review see Rogers 2014). Thus, there is strong evidence for hemispheric specialization in sensory processing across the animal kingdom.

Among sensory functions, olfaction has not attracted as much attention as other sensory systems while it may have specific characteristics of interest. Considered as an ancient or “primitive” sense, well conserved, and often presented as unilateral given the ipsilateral projections of the olfactory epithelium to the brain, it may retain original features of sensory lateralization. However, recent studies revealed more extensive connections between the olfactory bulbs (OB) than previously reported in some species (Yan et al. 2008; Grobman et al. 2018; Schaffer et al. 2018; Kermen et al. 2020) suggesting more inter-hemispheric interactions than commonly thought.

In the light of studies conducted in other sensory systems, we propose a descriptive state of the art of the literature regarding asymmetry in reception, sampling and processing of the olfactory stimulus, from which we can try to draw testable hypothesis to gain a better understanding of the function of olfactory lateralization. We first focus on the behavioral evidence of lateralized olfactory sampling, then we discuss asymmetric activations induced by odorant stimulation in relation to the anatomy of the olfactory pathway and to behavior.

## Behavioral evidence of lateralized olfactory processing

### Olfactory dominance: is there a best nostril?

Studies in humans revealed different perceptual performances when the odorant stimulus is experimentally delivered to the left or to the right nostril.

Measuring detection performance in humans, Toulouse and Vaschide reported a left nostril advantage for camphor detection in 56 out of 64 (86%) subjects including males, females, and children (Toulouse and Vaschide 1900). A more recent study reported no nostril advantage for amyl-acetate detection in a group of forty male and female right-handed subjects (Koelega 1979). In another work, right-handers performed better with their right nostril, whereas left-handers were more sensitive with their left nostril (Youngentob et al. 1982), suggesting a link between handedness and olfaction. Partially in line with this, Manescu et al*.* showed a right nostril advantage in eucalyptol detection in right-handers but no best nostril in left-handers (Manescu et al. 2017). However, testing detection of several different odorants in a large group of right- and left-handed subjects revealed no nostril advantage with no effect of handedness (Zatorre and Jones-Gotman 1990). Other authors reported no difference in detection thresholds between the two nostrils regardless of handedness (Betchen and Doty 1998). A confounding factor in these conflicting reports could be the ability of the stimuli to stimulate the trigeminal system, as it is the case for eucalyptol. The trigeminal system is a bilateral and crossed sensory system reporting on the pungent, irritant, or toxic component of odorant molecules and may interfere with olfactory responses (Frasnelli et al. 2008).

Regarding intensity rating, experiments carried out in humans revealed that the intensity of an odorant is evaluated as stronger when it is presented to the right nostril compared to the left (Thuerauf et al. 2008; Manescu et al. 2017). The same benefit of the right nostril has been observed in response to presentation of high concentration eugenol (Burne and Rogers 2002). Pendse et al*.* also reported a right nostril advantage in determining odorant concentrations, but in women only (Pendse 1987).

In a discrimination task, Hummel et al*.* reported that left- and right-handers showed, respectively a left and right nostril dominance (Hummel et al. 1998). A right nostril advantage was also described in males and females even though in this study, the relationships with handedness were weak (Zatorre and Jones-Gotman 1990). Right-handed human subjects were also found to be better at discriminating unfamiliar odorants using their right nostril. In contrast, if odorants were familiar scents, the success rate was the same for both nostrils (Savic 2000). An interpretation of these results involves an influence of the semantic dimension of the processing of olfactory information. Indeed, unfamiliar scents would be processed by the right hemisphere while when the subjects were familiar with the scents, they sought to name the scents and this would solicit the regions of language, located in the left hemisphere. Recently, in an odor identification task, a nostril advantage was reported in right-handed subjects, switching from the right nostril in younger subjects (< 18 years) to the left nostril in adults (Zang et al. 2020). Such switch from the right to the left hemisphere could possibly be linked to an enlargement of the sematic repertoire associated to odorants in adults compared to younger subjects, leading to a greater solicitation of the left hemisphere to identify odorants. The right nostril stimulation was also reported to elicit faster responses to a pleasant versus an unpleasant odorant in a task of affective judgment (Bensafi et al. 2002). However, no difference in the assessment of the pleasantness of odors according to the stimulated nostril was found (Thuerauf et al. 2008).

In birds, the first evidence of olfactory functional lateralization was found in chicks, by exploiting the phenomenon of maternal imprint to make the young memorize odorous stimuli (Vallortigara and Andrew 1994). This study uncovered that chicks performed better on an olfactory discrimination task between familiar and unfamiliar stimuli when the stimulations were delivered to the right nostril. Accordingly, an advantage for the right antenna in olfactory learning was reported in honeybees (Letzkus et al. 2006).

**Fig. 1:**
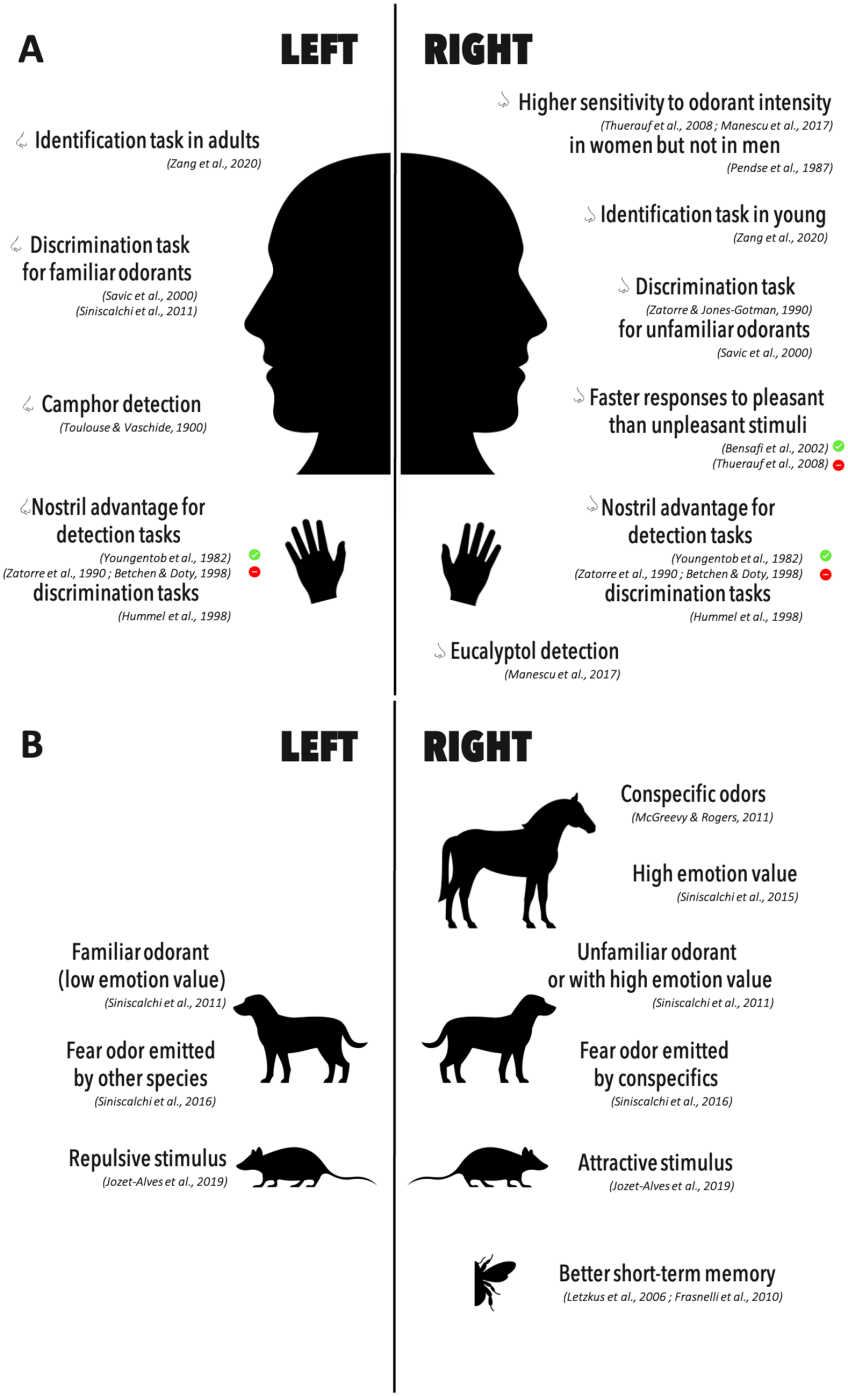
Left and right nostril asymmetries in human and non-human animal species. **A** Reported differences in nostril performances in humans, involving handedness or not. **B** Preferred nostril uses in non-human animal species. Red and green dots, respectively infirm and confirm the nostril advantage

In summary, nostril dominance has been studied principally in human. It appears to depend on several factors, such as the nature of the stimulus or the olfactory task, under investigation, manual preference, age, and gender, notwithstanding the effects of their combination. Nevertheless, given the predominantly ipsilateral connections of the olfactory epithelium to OB and piriform cortex, these data suggested that, when present, nostril asymmetry may reflect specialized olfactory information processing by the two sides of the olfactory brain, accounting for performance differences. In this context, and despite some discrepancies in the literature, an emerging picture designates the right nostril/hemisphere as best performing for detection and discrimination and left nostril/hemisphere as best performing for odor identification or odor detection and discrimination of already memorized stimuli like familiar ones (Fig. 1A).

### A preferred nostril according to the behavioral value carried by the olfactory stimulation

Pursuing on the comparison with other sensory modalities, it appeared from behavioral asymmetry in visual tasks that the two hemispheres process visual stimuli carrying specific behavioral significance, such as predator or food-related stimuli, as mentioned above. Thus, depending on the information borne by the scent, the two hemispheres would not be as effective in carrying out different tasks in olfaction. Consistent with this view, numerous experimental results, almost exclusively obtained in animal species (but sparse data were reported in humans, Millot and Brand 2000), revealed a lateralized sampling of the odorant depending on its ecological or emotional value. In other words, many species actively choose one or the other nostril for sampling the odorant.

Experiments conducted in dogs, using video recording of the position of the nostrils with regard to the odor source, revealed that they preferentially used their right nostril when facing unfamiliar stimuli with no aversive emotional value. Interestingly, if these stimulations were repeated and the scent became familiar, then dogs preferentially used their left nostril to sample the scent. On the other hand, if the stimuli used had an aversive emotional value (vet sweating, adrenaline supposed to activate the fear system, etc.), the dogs used their right nostril even when the exposure was repeated. Thus, in dogs, the olfactory system mobilizes the right nostril in the face of new, unfamiliar stimuli, and then a shift takes place towards the left nostril when the smell becomes familiar, provided that the latter has no negative emotional value (Siniscalchi et al. 2011). In a complementary study, the authors showed that, exposed to an odor emitted by an individual of the same species having experienced fear, dogs sample the stimulus with their right nostril. In contrast, the left nostril is used to smell an odor emitted by a human having experienced fear (Siniscalchi et al. 2016). So, beyond emotional value, the species from which the stimulus was isolated plays an important role in which nostril is used for scent sampling.

In other mammalian species as well, differences in use can be observed between the two nostrils. This is the case in horses for which the preferential use of one of the two nostrils was evaluated by presenting to the horse an odor of feces of congeners. The results showed a bias towards the preferential use of the right nostril to sample the scent (McGreevy and Rogers 2005). In addition, when the odors presented to horses carried emotional value, they preferentially used their right nostril to sample them. This is the case, for example, for the odors of adrenaline or female urine in estrus (Siniscalchi et al. 2015). Finally, in mice, attractive stimuli were reported to be sampled by the right nostril and less attractive stimuli by the left nostril (Jozet-Alves et al. 2019) (Fig. 1B).

Importantly, these behavioral data expanded the notion of best nostril describing better performance of one nostril over the other when a stimulus is delivered unilaterally by the experimenter, to that of preferred nostril, considering the decision made by the animal to use one nostril or the other based on the behavioral value of the stimulus. Although available data are too scarce to draw a coherent general scheme, a consistent finding in dogs and horses was the active sampling by the right nostril for unfamiliar or intraspecific aversive stimuli. Further and more systematic investigation of the preferred nostril would be useful in humans and diverse animal species to extract the common features of the stimuli actively explored with either side of the olfactory system and get a better picture of hemispheric specialization in odorant processing. In this context, it would be relevant to assess sampling biases for biological odorants and for odorants to which learning would have assigned a behavioral or emotional significance.

It follows from the existence of a preferred nostril that olfactory–motor processes have to be modulated in a top-down and asymmetric manner, shortly during the first sampling of the odorant to implement the nostril choice made by the animal. Motor adjustments may include head orientation, as in the chick turning its head to select one eye or the other according to the visual stimulus, and nose twitching. Such top-down signals are triggered by temporal shifts and intensity differences in the stimulus reaching the sensory organs (Rajan 2006; Louis et al. 2008; Esquivelzeta Rabell et al. 2017; Liu et al. 2020) and are at play in fast nose orientation towards a laterally positioned source of odorant (Esquivelzeta Rabell et al. 2017). Whether they contribute to nostril choice remains to be investigated. In addition, one could hypothesize asymmetric modulation of the sniff amplitude or frequency. Indeed, sniffing is a highly regulated olfactomotor behavior. It is modulated by familiarity and learned significance of the odorant in rodents (Lefèvre et al. 2016) or by the hedonic value of the odorant in humans (Bensafi et al. 2003). In addition, sniff modulation can occur within less than 200 ms (Johnson et al. 2003). However, although not fully identified, the brain circuit underlying olfacto-motricity is closely related to that controlling respiratory muscles and there is no evidence yet that it would be able to implement an asymmetric control of sniffing.

## Anatomical or functional asymmetry of the peripheral olfactory organs

### The nasal cycle

An obstacle to interpreting any superiority of one nostril over the other or the choice of the preferred nostril is the physiological alternance that occurs in the odorant-carrying airflow between the two nostrils. Indeed, in humans, nostrils are alternatively congested, leading to reduction of the airflow entering the nostrils. The period of the nasal cycle was reported to be of about 2 h for one nostril (Kahana-Zweig et al. 2016). Similarly, in rodents, a nasal cycle with a period of 30–85 min was reported (Bojsen-Moller and Fahrenkrug 1971) (but see also a more recent work using pressure sensors that failed to measure any nasal airflow rhythm in rat (Parthasarathy and Bhalla 2013). Consistent with the alternance of nostril congestion and reduction in the nasal airflow, an early study using 2-deoxyglucose up-take to map neuronal activation found various degrees of asymmetric activation of the left and right olfactory bulbs with no preference for either side in about two-third of the tested rats while one-third showed symmetric bulbar activation (Stewart et al. 1979). These data suggest that the nasal cycle may interfere when measuring the performances of the two nostrils. However, the nasal cycle is not synchronized and, over large groups of subjects or animals, would be more likely to dampen rather than account for the better performance of one or the other nostril or for active choice of one nostril to sample the stimulus.

### Peripheral morphologic or functional differences

Nostril dominance may arise from peripheral morphologic or functional differences between the two sides of the nasal cavity or the olfactory epithelium.

In humans, nasal cavity asymmetry measurements have been carried and reported mostly in pathologies (James et al. 1991; Freeman et al. 2013) and asymmetry in nasal cavity may be of low significance in healthy subjects. On the functional aspect, a main source of asymmetry could relate to olfactory sensory neurons (OSN) and/or olfactory receptors distribution introducing differences in sensitivity or selectivity. While the spatial pattern of olfactory receptors expression within the olfactory epithelia is well described in humans and rodents (Mori et al. 2000), comparisons between the two sides have been less documented yet to the best of our knowledge. This would be a step to take to elucidate a possible peripheral origin of nostril dominance for some odorants.

In other animal species, especially in non-vertebrate, research has been more intensive. In honeybees, Letzkus et al. asked if the right antenna advantage in olfactory learning could arise from asymmetry in reception between the two antennae (Letzkus et al. 2006). Olfactory receptors named sensilla placodea were counted on the bee antennae using scanning electron microscopy and proved to be 10% more numerous on the right compared to the left antenna. However, only a part of the antenna was analyzed and only one type of sensilla was counted. Nevertheless, Frasnelli et al. confirmed these observations in the honeybee species *Apis mellifera*: the right antenna was better than the left in olfactory short-term memory (< 1 h) and contained more putative olfactory sensilla (placodea, trichodea and basiconica), whereas the left antenna contained more sensilla not involved in olfaction (campaniformia, coeloconica and chaetica) (Frasnelli et al. 2010). Furthermore, electrophysiological responses showed a higher responsiveness of the right compared to the left antenna to olfactory stimuli (Anfora et al. 2010). This asymmetric repartition of sensilla between the antennae could modulate the reception of the stimulus and account for the difference in learning and memory performances using one or the other antenna. A right antenna advantage in olfactory learning has then been reported in other Hymenoptera species, such as the bumblebee species *Bombus terrestris* (Anfora et al. 2011), three different stingless honeybee species *Trigona carbonaria*, *Trigona hockingsi* and *Austroplebeia australis* (Frasnelli et al. 2011), and in female wasps *Anastatus japonicus* (Meng et al. 2012).

In *Caenorhabditis elegans* (*C. elegans*), olfactory asymmetry was described at the odorant receptor level. In *C. elegans*, the number of neurons is relatively limited: 302 neurons out of 952 somatic cells and among them, 32 are chemosensory neurons. These chemosensory neurons drive attractive or repulsive behaviors to different stimuli. Among these stimuli, volatile molecules elicited responses from AWA, AWB and AWC neurons: AWA and AWC neurons responded to attractants, whereas AWB neurons responded to repellants (Bargmann et al. 1993; Troemel et al. 1995). The AWC neurons are of special interest when considering asymmetric repartition of olfactory receptors. Indeed, Troemel et al*.* showed, using the expression of a fluorescent *str-2* transgene, that in adult nematodes, only one of two morphologically identical AWC neuron expressed the gene *str-2*, either the left or right one (Troemel et al. 1999). Surprisingly, this asymmetry of *str-2* expression appeared to be stochastically allocated to the left or right AWC neuron, raising the question of its physiological role. Indeed, the *C.elegans* mutant ky542, expressing *str-2* on both sides exhibited degraded olfactory perception (Wes and Bargmann 2001). The question of the possible existence of other such genes that may be asymmetrically expressed is open. If it is the case, are their asymmetric expression co-regulated? Do they allow a precise asymmetric olfactory receptor organization and what are the behaviors regulated on the basis of the asymmetric expression of olfactory receptors? Still in *C. elegans*, ASE neurons are other chemosensory neurons detecting soluble attractants (Bargmann et al. 1993). They asymmetrically express a guanylyl cyclase thought to be a chemosensory receptor detecting non-volatile chemicals. Nonetheless, this asymmetric expression is non-stochastic and appears strictly regulated: two members of the guanylyl cyclase family are expressed in the left ASE neuron while one other member is expressed in the right ASE neuron (Yu et al. 1997). Asymmetric distribution of olfactory receptors in non-vertebrates may provide interesting models to assess the detailed neural connectivity of the lateralized neurons and decipher how such asymmetric circuits drive odorant approach or escape behaviors, inspired by the way other sensorimotor mechanisms were dissected out in non-vertebrates (Jovanic et al. 2016). In vertebrates, the spatial distribution of several identified olfactory receptors in selected zones of the olfactory epithelium was recently described in mice and showed an overall symmetrical expression between the two sides of the olfactory epithelium (Zapiec and Mombaerts 2020). This report does not support a differential olfactory receptors expression between the two sides of mammalian olfactory mucosa as the potential basis for nostril dominance and lateralized processing of olfactory signals but rather points towards asymmetry originating from central processing.

## Asymmetric central processing of olfactory inputs

### Insights from anatomy

In mammals, the ascending olfactory projections are ipsilateral from the olfactory epithelium to the OB and piriform cortex, but there are several levels of interhemispheric interactions allowing olfactory signal sharing between the two hemispheres. OSN project onto the relay neurons, the mitral/tufted cells of the ipsilateral OB within neuropils called glomeruli (Pinching and Powell 1971; Mori 1999). OSN expressing the same olfactory receptor innervate a set of glomeruli which in turn receive inputs only from one type of molecular receptor (Mombaerts et al. 1996). As a result, any odorant elicits a stereotyped spatial pattern of activated glomeruli reflecting the tuning of the sensory neurons. This activation map was described as symmetrical in the left and right OB, at least at the macroscale of glomeruli positions (Sullivan et al. 1995; Mori and Sakano 2011). The two OB maps are topographically connected to each other by an indirect pathway involving bilaterally the outermost region of the anterior olfactory nucleus pars externa (AONpE) (Yan et al. 2008). Interestingly, AONpE can be activated by a stimulation applied to the ipsilateral nostril and inhibited by a contralateral nostril stimulation and this integrative property may sub-serve odorant source localization (Kikuta et al. 2010). In addition, unilateral optogenetic stimulation of the OB was found to elicit mirror activation maps in the contra-lateral OB (Grobman et al. 2018). Further investigations confirmed that AONpE projects to the iso-functional mirror-symmetric mitral-tufted cells of the contralateral OB through excitatory glutamatergic mechanisms. A computational approach suggested that the contra-lateral odorant representation contained information allowing for odor identity decoding (Grobman et al. 2018; Dalal et al. 2020). Thus, this inter-bulbar circuit would allow sharing of olfactory afferent information between the two OB. The OB then projects ipsilaterally to the piriform cortex and left and right piriform cortices exchange information through the anterior commissure. In the rodent piriform cortex, following unilateral stimulations, neurons can be found with receptive fields in the right or left epithelia as well as neurons responding to bilateral stimulations (Wilson 1997). The anterior commissure allowing transferring olfactory information from one side to the other of the piriform cortex could contribute to the emergence of these contralateral or bilateral receptive fields of piriform cortex neurons. Bilateral processing of olfactory signals by the piriform cortex could be involved in spatial navigation. Indeed, in pigeons, who used olfactory indices for traveling back to their home position, lesions of the left piriform cortex impaired navigation. Birds were transported to an unfamiliar position and released to allow them to come back home. Those with a left piriform cortex lesion took scattered directions while those with a right piriform cortex lesion headed to the correct direction, as non-lesioned controls did (Gagliardo et al. 2005). Interestingly, although a similar specialization of the piriform cortex in spatial navigation is not described to the best of our knowledge in mammals, the left and right hippocampi, to which the piriform cortex in connected through the entorhinal cortex, govern distinct mode of spatial navigation in human with the left hippocampus activated in egocentric navigation (versus the right hippocampus in allocentric navigation) (Iglói et al. 2010). Finally, in rat pups younger than 12 days, an olfactory memory trace is formed in each hemisphere following unilateral stimulation and retrieval of this memory by the contralateral naris requires the later development of the anterior commissure (Kucharski et al. 1986). Thus, communication between the two sides of the piriform cortex through the anterior commissure could also be used for bilateral memory retrieval.

In the zebrafish, projections from the epithelia are unilateral and ipsilateral but direct reciprocal connections between the OB were recently demonstrated (Kermen et al. 2020). Mitral cells were found to be directly connected between the two OBs, with preserved topographical organization, allowing for communication between homologous olfactory columns. These inter-bulbar communications increased the detection of pheromones. In *Drosophila*, OSN located in the olfactory epithelium project to both the left and right sides of the brain (Stocker et al. 1990). Despite this bilateral structural connectivity, Gaudry et al*.* showed that at the functional level, OSN released about 40% more neurotransmitter to their ipsilateral than to their contralateral target. In turn, in response to an asymmetric odor stimulation, the ipsilateral central neurons began to fire slightly earlier than contralateral central neurons. These functional asymmetries of odor processing result in a lateralized behavior in which the *Drosophila* turns towards the odor source, i.e. to the side of the antenna that catches the odorant (Gaudry et al. 2013).

Thus, in most species, the olfactory system projects ipsilaterally. However, the afferent message reaches the OB bilaterally and symmetrically, although, unlike other sensory system in which the afferent information is directed to both sides of the brain, olfactory information follows an indirect path to the contralateral side. In mammals, it goes through a multi-synaptic pathway through the AON. Therefore, the contralateral representation is attenuated (Yan et al. 2008) and is likely to be delayed along the multi synaptic circuit. One may hypothesize that both phenomena will lead to an asymmetric odor representation from the early stage of olfactory processing, especially in case of asymmetric odor sampling.

### Odorant-evoked asymmetric brain activity

In humans, asymmetry in odor-evoked brain responses was first described using PET brain imaging in the right orbitofrontal cortex which exhibited higher level of activation than its left counterpart in response to olfactory stimulations (Zatorre et al. 1992). This finding was consolidated by several fMRI studies and appeared consistent with the best performance of the right nostril reported in odor detection as discussed above. However, higher right orbitofrontal activation occurred regardless of the stimulated nostril. In addition, detection tasks tended to be associated with a lower level of lateralization than identification tasks, suggesting that deeper cognitive processes were associated with enhanced asymmetry. Piriform cortex and orbitofrontal cortex were also asymmetrically activated by odor recognition tasks and familiarity judgements with a stronger involvement of the right hemisphere (reviewed in Royet and Plailly 2004). These latter data do not appear consistent with the left nostril advantage described by some authors in an identification task (Zang et al. 2020) and will need to be clarified. Asymmetric brain responses were also reported for hedonic judgements, pleasant odorants producing larger responses in the left hemisphere and negative value odorants in the right hemisphere, promoting the view of dissociated pathways along the left or right hemisphere for positive or negative hedonic odorant values, respectively. Asymmetric responses included those of the orbitofrontal cortex, amygdala (Patin and Pause 2015) and piriform cortex (Zelano et al. 2007). Partially conflicting results were also reported for amygdala, showing a predominant left activation in response to unpleasant odorants and bilateral balanced activation in response to pleasant odorants (Gottfried et al. 2002). It was further suggested that this previous finding could have been obscured by an effect of odorant concentration (Anderson et al. 2003). Here again, the link with the best or preferred nostril is unclear. Indeed, in humans, hedonic judgements were reported to be unaffected by the side of the stimulation although processed faster through the right nostril (Bensafi et al. 2002; Thuerauf et al. 2008). In mice, asymmetry in odorant sampling suggested a lateralization pattern opposite to what is proposed in humans, with attractive odorants sampled by the right nostril and vice versa (Jozet-Alves et al. 2019) but no data on brain responses are available yet. In rat, the response of the piriform cortex was analyzed along the course of an olfactory-associative training and revealed an asymmetric pattern of the Beta and Theta oscillations between the left and right piriform cortexes that evolve with the stages of learning (Cohen et al. 2015). Most interestingly, these effects were retrieved in successful trials compared to errors trial supporting a role of the asymmetry in performance. Dynamic asymmetry in the orbitofrontal cortex, a secondary olfactory brain structure, was also reported during a reversal learning task (Cohen and Wilson 2017). Thus, olfactory representations in the piriform and orbitofrontal cortex show dynamic asymmetry associated to the acquisition of an olfactory task, pointing to a functional role of lateralized olfactory processing in learning.

To sum up, asymmetries were reported regarding odor-evoked brain activation in basal condition and during olfactory learning in a task and performance manner. However, before more systematic investigations of the effect of the stimulation side on brain lateralization in different types of olfactory tasks, it is hazardous to draw any conclusion regarding the extent to which the best or preferred nostril determines the lateralized activation in the olfactory pathway and beyond. Furthermore, intrinsic properties of cortical circuits may also account for asymmetric processing of sensory information. No data are yet available on the olfactory cortex but in audition, in the left and right auditory cortexes for instance, microcircuits are organized to process distinct spectro-temporal features of sounds, some of them potentially related to biological significance such as vocalizations (Levy et al. 2019). The analogy with odorant molecule features remains to be investigated. Some physico-chemical properties of odorants governing perception would be good candidates, such as molecular complexity, which is correlated to hedonics in humans and animals (Khan et al. 2007; Kermen et al. 2011).

Besides activation levels and oscillatory activities, afferent stimulation may drive changes in connectivity within or between hemispheres. In rat, using local field potential recordings, olfactory learning proved to first induce a decrease in temporal coherence between the left and right piriform cortexes during the initial phase of learning recovering as the animals became experts in the task (Cohen et al. 2015). Changes in intra-hemispheric connectivity between the piriform and orbitofrontal cortices were also observed in relation to the stages of olfactory learning (Cohen and Wilson 2017). A better understanding of the asymmetric functioning of the olfactory brain will then require to assess not only brain activation levels but also brain connectivity in future studies. Interestingly, in humans, asymmetry in evoked-related potentials between the two hemispheres was shown to be reduced in culinary experts compared to control subjects during olfactory mental imagery suggesting an effect of experience on inter hemispheric functional connectivity (Bensafi et al. 2017).

## Conclusion

Lateralized patterns of brain responses to odorant stimulations were reported, mostly in humans. They were obtained using multiple olfactory tasks and stimuli and this might be a reason why it is currently difficult to gain a global understanding of their functional significance. In particular, the level of familiarity, the hedonic value and olfactory learning may interfere with olfactory brain lateralization in terms of activity and connectivity but no general rule relating the nature of the task to brain lateralization could be enacted. Numerous behavioral evidences suggested that olfactory brain lateralization may arise from peripheral asymmetry. This view is supported by asymmetry in perceptive performances varying according to the stimulated nostril. A best nostril has been described in all species that were investigated, the left or the right one, depending on the type of odorants or tasks. Considering the dominant ipsilateral organization of the olfactory pathway, these data were interpreted as reflecting hemispheric specialization of olfactory information processes. However, the relationships between the best nostril and lateralization of brain patterns remained unclear. We argue that the asymmetry in olfactory sampling, which represents animal decision on which side of the brain should be privileged for a given stimuli, is of particular interest in this perspective. It is an active process, conserved over the phylogeny, involving olfactomotor control and dependent on the olfactory tasks to be accomplished, as suggested by current data. Asymmetry in odorant sampling may be of strong ecological pertinence and as such might prove to be functionally related to the lateralized brain patterns, a hypothesis that remained to be tested. Hemispheric specialization in olfactory processing could also arise in a non-exclusive manner from distinct properties of the sensory organs of the two sides of the body, organization of the sensory pathway or intrinsic properties of cortical circuits of the left and right hemispheres. Better knowledge on the regulation and function of brain lateralization may help the understanding of neurodevelopmental or psychiatric pathological conditions showing altered brain lateralization (Kleinhans et al. 2008; Anderson et al. 2011; Wachinger et al. 2018; Wiberg et al. 2019). To achieve this, the rodent olfactory system, well conserved along the phylogeny, in which the sensory information fed to the brain can be controlled and brain network manipulated, may be a model system of interest for further studies.
